# Expression and purification of the mammalian translocator protein for structural studies

**DOI:** 10.1371/journal.pone.0198832

**Published:** 2018-06-13

**Authors:** Elisabeth Graeber, Volodymyr M. Korkhov

**Affiliations:** 1 Laboratory of Biomolecular Research, Division of Biology and Chemistry, Paul Scherrer Institute, Villigen, Switzerland; 2 Institute of Biochemistry, ETH Zurich, Switzerland; University of Bern, SWITZERLAND

## Abstract

The translocator protein (TSPO) is an 18 kDa polytopic membrane protein of the outer mitochondrial membrane, abundantly present in the steroid-synthesising cells. TSPO has been linked to a number of disorders, and it is recognised as a promising drug target with a range of potential medical applications. Structural and biochemical characterisation of a mammalian TSPO requires expression and purification of the protein of high quality in sufficiently large quantities. Here we describe detailed procedures for heterologous expression and purification of mammalian TSPO in HEK293 cells. We demonstrate that the established procedures can be used for untagged TSPO as well as for C-terminally fused TSPO constructs. Our protocol can be routinely used to generate high-quality TSPO preparations for biochemical and structural studies.

## Introduction

The translocator protein (TSPO) is an 18kDa protein originally discovered as a secondary target of diazepam [1, 2], hence one of its earlier names, peripheral benzodiazepine receptor (PBR). TSPO is widely conserved across species from bacteria to mammals. In humans it is found in most tissues, including adrenal, kidney, heart, and platelets [3], and especially in steroid-synthesising cells where TSPO is enriched in the outer mitochondrial membrane (OMM) [4]. TSPO is mainly found at inner-outer membrane contact sites and has been suggested to be part of the mitochondrial permeability transition core complex, along with the voltage dependent anion channel (VDAC) and the adenine nucleotide transporter (ANT) [5]. In mammals, TSPOs main function has been hypothesised to be the translocation of cholesterol through the OMM, a process which is probably carried out not by TSPO alone, but in cooperation with one or several other proteins [6]. One leading hypothesis regarding how this process is carried out involves delivery of cholesterol to the membrane by the steroid acute regulatory protein (StAR); once delivered, cholesterol is taken up by TSPO, which then translocates cholesterol across the OMM to the IMM. The precise mechanism of translocation is not known, but might involve either passage of cholesterol through a channel-like cavity within TSPO, or it could occur at the protein-lipid or protein-protein interface, which may involve TPSO and other components of the OMM. Cholesterol is thereby enriched in the IMM and is passed over to the P450scc, which in turn mediates the conversion of cholesterol to pregnenolone, the first and rate-limiting step of steroidogenesis [7, 8]. It has long been suggested that TSPO may play a key role in regulating this step by controlling the rate of cholesterol influx to the IMM [9, 10]. Recent reports involving TSPO knock-out mice have cast doubts on the key role of TSPO in the steroid production [11, 12]. It remains to be seen whether in healthy wild type cells TSPO plays a canonical role as a master regulator of steroid synthesis proposed many years ago, or whether its physiological function is different.

Notwithstanding the open questions about its true molecular function in the cell, the interest in TSPO as a pharmacological target has steadily increased since its recognition as a drug target for benzodiazepines. Changes in TSPO expression levels have been associated with a number of diseases [13]. For example, TSPO is up-regulated in various neurological disorders, including: Alzheimer’s [14], multiple sclerosis [15], amyotrophic lateral sclerosis, Huntington’s disease, and Parkinson’s [16]. Overexpression of TSPO has been linked to the degree of damage in central and peripheral nervous tissues [17, 18]; as a consequence TSPO is used as an imaging tool for traumatic brain injury [19]. TSPO has been shown to be down-regulated in a range of psychological conditions [20]. TSPO is down-regulated in anxiety disorders, such as generalised anxiety [21] and social anxiety disorder [22] posttraumatic stress disorder [23], and schizophrenia [24]. A polymorphism in the TSPO gene, 485G>A, results in higher susceptibility of the patients to panic disorder [25]. Beyond its relevance to neuropharmacology, TSPO has been reported to be overexpressed in some forms of cancer [26, 27], and expression of TSPO has been shown to correlate to the aggressiveness of the tumour [28]. This underscores the high value of TSPO as a diagnostic and therapeutic target in a range of human pathologies.

TSPO is composed of five alpha helices embedded in the membrane, with only small inter-helical loops extending into the mitochondrial inter-membrane space or into the cytosol. The helical arrangement was confirmed by our cryo-EM-based reconstruction of the *R*. *sphaeroides* TSPO at 10 Å resolution, which revealed a dimer of TSPO molecules with each monomer composed of five TM domains [29]. A number of high-resolution structures of TSPO have recently been solved, including X-ray structures of two bacterial TSPO homologues (*B*. *cereus* and *R*. *sphaeroides*) and an NMR structure of the mouse TSPO [30–32]. While the structures of bacterial TSPO homologues show a high degree of similarity, the mouse TSPO appears to have substantial structural differences [33]. While the overall fold of the five TM domains in the NMR structure is similar, several of the helices appear to be significantly shifted and twisted, compared to similar elements in the bacterial TSPO structures. This suggests that the single structure of a mammalian TPSO homologue available to date may not be sufficient to understand the protein in its active state, for reasons that were discussed in detail elsewhere [33]. A high-resolution structure of a mammalian TSPO homologue in its functional form would shed light on the key questions pertaining to the function of the TSPO family of proteins.

The lack of a high-resolution structure of a mammalian TSPO, other than the available NMR structure, can to some extent be attributed to the difficulties in overexpression and purification of membrane proteins in their functional form [34]. Although in many instances it is possible to overproduce mammalian membrane proteins in prokaryotic hosts, this often leads to generation of non-functional, aggregated or misfolded protein preparations [35, 36]. Expression in eukaryotic cell hosts, such as yeast, insect or mammalian cells is often beneficial for correct folding and preserved functionality of the membrane protein of interest [37, 38]. The task of generating membrane protein preparations of high purity for structural studies is further complicated by the necessity of generating milligram quantities of material for downstream processing and analysis (this is true for X-ray crystallography, NMR and cryo-EM). Membrane proteins are often present at low concentration in the cell. Although some of the naturally abundant membrane proteins can be isolated from native sources, this is not always possible [39]. Membrane proteins are confined to a limited available space in the cell membranes, and can be subject to complex targeting events during an overexpression experiment.

To overcome the difficulties with obtaining a high-resolution structure of TSPO in a physiologically relevant state, it is necessary to establish a procedure for producing a high-quality purified sample of the protein. Here we describe an optimised protocol to obtain homogenous, stable and functional protein in quantities sufficient for functional and structural studies.

## Materials and methods

### Reagents

All chemicals used in buffer preparations were from Gerbu, except imidazole, which was from Merck. LB broth for small-scale bacterial cultures was from Conda. Mammalian cell culture media, DMEM and PEM, were from BioConcept and Thermo Fischer Scientific, respectively. Fetal Calf Serum was purchased from Seraglob. Penicillin-streptomycin-amphotericin B (PSA) and Glutamax were from Gibco. Detergents, including dodecylmaltoside (DDM), decyl maltoside (DM), lauryldimethylamine oxide (LDAO), C_12_E_8_, octyl glucoside (OG), nonyl glucoside (NG), octyl maltoside (OM), nonyl maltoside (NM) and octyl thioglucopyranoside (OTG) were purchased from Anatrace (all detergents were ana-grade; DDM used for solubilisation was sol-grade). HisPur Cobalt resin and StrepTactinXT Superflow resin were purchased from Thermo Fischer Scientific and iba, respectively. Cholesteryl hemisuccinate, 25 kDa branched PEI, sodium butyrate and tetracycline were from Sigma. Biotin was purchased from Fluorochem. Radioligand [^3^H]PK11195 was from PerkinElmer.

### Cloning

Twelve TSPO homologues were cloned into pCDNA3.1, including: human, mouse, rat, dog, bovine, pig, sheep, chicken, *xenopus laevis*, *danio rerio*, *danaus plexipus* and *drosophila melanogaster*. All constructs were tagged by a N- or a C-terminal GFP and a 10xHis tag (24 constructs in total). For stable cell line generation the constructs were cloned into pACMV-tet-o [40] containing a C-terminal YFP and a 10xHis tag, separated from TSPO by a 3C protease cleavage site.

### Small-scale expression and detergent screen

All constructs were expressed in HEK293T cells by transient transfection using 25 kDa branched PEI, following an established procedure [41]; HEK293T cells were from by K. Ballmer-Hofer (Paul Scherrer Institute). Cells were harvested after 48 h and spun down at 1000 g, 5 min, 4°C. Cell pellets from one 10 cm plate were resuspended in PBS, split into 6 aliquots, spun down again at 1000 g, 5 min, 4°C and frozen at -80°C until the day of experiment.

Pellets were resuspended in 50 mM Tris (pH 7.5), 200 mM NaCl and supplemented with Complete EDTA-free protease inhibitor cocktail (Roche) and 0.5 mM PMSF. Cells were disrupted by sonication and detergent was added for solubilisation (1% final detergent concentration). After 1 h, the insoluble material was removed by ultracentrifugation (40k rpm, 30 min, rotor TLA 100.3) and the supernatant analysed by fluorescence size exclusion chromatography (FSEC) using the Agilent SEC5-300 column (flow 0.3 ml/min, excitation 488, emission 510). Two detergent screens were performed. First, the constructs were tested using mild detergents to find the most stable homologues. In a second screen, the most stable constructs were tested in small-micelle detergents, which, due to their small micelle size, may be favourable for potential subsequent crystallisation trials. For the first detergent screen, all C-terminally tagged constructs were tested with DDM, DM, LDAO and C_12_E_8_, while the N-terminally tagged construct were only tested with DDM and LDAO. Detergent concentration in the equilibration buffer was as follows: 0.02% DDM, 0.1% LDAO, 0.1% DM or 0.01% C_12_E_8_. For the second detergent screen, the remaining constructs were tested with small-micelle detergents. For this screen, solubilisation was carried out in 1% DDM, but after clearing the lysate by ultracentrifugation the sample was diluted 10-fold into a buffer with a secondary detergent: OG (1.2%), NG (1.0%), OM (0.5%), NM (0.5%), or OTG (0.5%). All samples were subjected to an additional ultracentrifugation step prior to FSEC analysis as described above, using SEC5-300 column equilibrated in a buffer containing 50 mM Tris (pH 7.5), 200 mM NaCl, 0.02% DDM.

### Radioligand binding assays

Cell pellets were resuspended in PBS and supplemented with Complete EDTA-free protease inhibitor cocktail (Roche) and 0.5 mM PMSF. Cells were homogenised by sonication for 15 sec using a rod sonicator (Sonics Vibra cell VCX 600, 0.5 sec on, 0.5 sec off, microtip, 40%). Total protein concentration was determined with a Bradford assay (BioRad). For the radioligand binding assay, a total protein concentration of 0.25 mg/ml was used. The radioligand [^3^H]PK11195 was used at a final concentration of 20 nM. Reactions were incubated on ice for 1 h. Glass fibre filters (0.4 μm GF/B, Whatman) were pre-soaked in 0.1% polyethyleneimine and then transferred to the 12-well filtration apparatus (Millipore). Filters were washed with 1 ml PBS. The binding reactions were stopped by vacuum filtration, followed by washing with 6 mL PBS. The filters were placed into scintillation vials, 6 mL liquid scintillation cocktail (LabLogic) was added into each vial, and the radioactivity trapped on the filters was counted using a Tri-Carb 3100 TR liquid scintillation analyser (Packard). Results of three experiments were plotted using GraphPad Prism version 7.00, GraphPad Software, La Jolla California USA, www.graphpad.com.

### Thermostability assays for homologue selection

To assess the stability of the solubilised TSPO, we used a previously described procedure with minor modifications [42]. In brief, the TSPO was expressed and solubilised as described above for the detergent screen. After ultracentrifugation, the supernatant was heated in a heat block to 27°C, 42°C, 50°C and 60°C for 1 h. Control samples were left at room temperature (22°C) and at 4°C. All samples were cleared by ultracentrifugation (Beckman Coulter Optima MAX, TLA 100.3, 40k rpm, 20 min, 4°C) and FSEC analysis was carried out as described above. The height of the protein peaks was used for plotting against the incubation temperature, and for determining the apparent melting temperatures (T_m_) of the protein in various tested conditions. The thermostability assay was performed with the homologues that were deemed as potentially promising after the detergent screen (human, mouse, rat, dog, bovine, pig, sheep, chicken and *Xenopus laevis*).

### Stable cell line generation

Stable cell lines were generated for pig and bovine TSPO, following a previously described procedure with minor modifications [43]. For each construct 24 clones were isolated; the TSPO expression levels in each of the clones were assessed using a small-scale solubilisation and FSEC. The best clones, judged by the magnitude and the quality of the FSEC peak, were used for further large-scale expression in suspension.

### Large-scale expression

Stably transfected HEK293S GnTi^-^ cells [40] were grown in suspension in a final volume of 3–8 L of media (PEM, 5% FCS, PSA, Glutamax). Upon reaching the cell density of ~3x10^6^ cells/ml, expression was induced with 2 μg/ml tetracycline and cultures were supplemented with sodium butyrate (5 mM). Cells were harvested after 72 h by centrifugation at 2500 g, FIOS 6x500y rotor (HiCenXL), 20 min, 4°C, frozen in liquid nitrogen and stored at -80°C until purification.

### Large-scale purification of untagged pig TSPO

Cell pellet (~7 g/L suspension culture) was resuspended in a buffer containing 50 mM Tris (pH 7.5), 200 mM NaCl, 10% glycerol, 5 ug/ml DNAse, supplemented with Complete EDTA-free protease inhibitor cocktail (Roche) and 0.5 mM PMSF. Cell walls were broken using a Dounce homogeniser and spun down by ultracentrifugation at 35k rpm, 1 h, 4°C (Ti45). The membrane pellet was resuspended, frozen and stored at -80°C until purification.

Membranes were resuspended in 50 mM Tris (pH 7.5), 200 mM NaCl, 20 mM imidazole, 10% glycerol. 0.5 mM PMSF, 5μg/ml DNAse and Complete EDTA-free protease inhibitor cocktail (Roche). Pellet was homogenised with Ultra-Turrax homogeniser. Solubilisation was carried out by adding 1% DDM and stirring for 1 h. Insoluble material was removed by centrifugation (4°C, 19,000 rpm, 30 min, rotor A8.24). Supernatant was added to HisPur Cobalt resin, incubated for 1 h with rotation and applied to a gravity column. The flow-through was reapplied and subsequently two wash steps were carried out using buffers containing 50 mM Tris (pH 7.5), 200 mM NaCl, 0.02% DDM, 10% glycerol and either 25 mM (first wash) or 50 mM (second wash) imidazole. The protein was eluted with a buffer containing 250 mM imidazole. The buffer was exchanged to 20 mM Tris (pH 7.5), 200 mM NaCl, 25 mM imidazole, 0.02% DDM, 10% glycerol by desalting using a HiPrep 26/10 column by FPLC at a flow rate of 5 ml/min. Fluorescent protein tag was removed by 3C protease cleavage overnight at 4°C. The 3C protease, the cleaved fluorescent protein tag, and any uncleaved TSPO fusion protein were removed by reverse IMAC purification with a pre-equilibrated Co-affinity column. The reverse IMAC flow-through fractions were concentrated with an Amicon concentrator (MWCO 30,000 kDa) at 2300 g, 4°C and protein was further purified by size-exclusion chromatography (SEC) using a Superdex200 Increase column pre-equilibrated in a buffer containing 20 mM Tris (pH 7.5), 200 mM NaCl, 0.5 mM EDTA, 10% glycerol, 0.02% DDM.

### Large-scale purification of pig TSPO fusions

Purification of the fusion construct follows a similar procedure as that for the untagged TSPO, with several key differences. Solubilisation was carried out by adding 1% DDM and 0.2% CHS. The supernatant was added to strepXT resin (3^rd^ generation) and incubated for 2 h. The resin bound was applied to a gravity column and washed with 25 column volumes of 50 mM Tris (pH 7.5), 200 mM NaCl, 0.5 mM EDTA, 0.02% DDM, 0.004% CHS. Elution buffer contained 50 mM Tris (pH 7.5), 200 mM NaCl, 0.5 mM EDTA, 0.02% DDM, 0.004% CHS, 50 mM biotin. The eluted sample was concentrated using an Amicon concentrator (MWCO 50,000 kDa), 2200 g, 4°C. Protein was further purified by SEC using a Superdex200 Increase column pre-equilibrated in a buffer containing 20 mM Tris (pH 7.5), 200 mM NaCl, 0.5 mM EDTA, 0.02% DDM and 0.004% CHS.

### SDS-PAGE

Expressed and purified proteins were analysed by SDS-PAGE. TSPO tagged with fluorescent protein tags was visualised by in-gel fluorescence using the Amersham Imager 600 from GE (settings: EPI_RGB-blue-Cy2). TSPO samples purified in large scale were analysed by staining the SDS-PAGE gels with Coomassie blue (Gerbu Brilliant Blue R #9152.0100).

### Tryptophan fluorescence quenching assay

Intrinsic tryptophan fluorescence quenching assays were performed in black greiner 96 well plates using the PHERAstar FSX plate reader at 28°C with the optical model FL ex280 em340. The final protein concentration was 1 μM and the ligand concentration 0–50 μM. The sample volume was 120 μl/well; the ligand stocks were made in DMSO and the reactions were performed in 20 mM Tris pH 7.5, 200 mM NaCl, 0.5 mM EDTA 0.02% DDM, 0.004% CHS. Samples were incubated at room temperature for 10 min before the measurement. All measurements were performed in triplicates. Apparent K_d_ values were calculated using non-linear regression in GraphPad Prism 7.0.

### Thermostability assays of purified TSPO with ligands

Purified pig TSPO-YFP was incubated at 60°C for 1 h, in the absence or in the presence of the ligands (100 μM): PK11195, diazepam, protoporphyrin (PPIX) and NBD-cholesterol. A fluorescent analogue of cholesterol, NBD-cholesterol, was chosen based on its higher solubility compared to cholesterol. Upon incubation, samples were cleared by centrifugation (25,000 rcf, 40 min, 4°C) and the supernatants were analysed by FSEC as described above.

## Results

### Identification of a promising TSPO homologue

**Fig 1:**
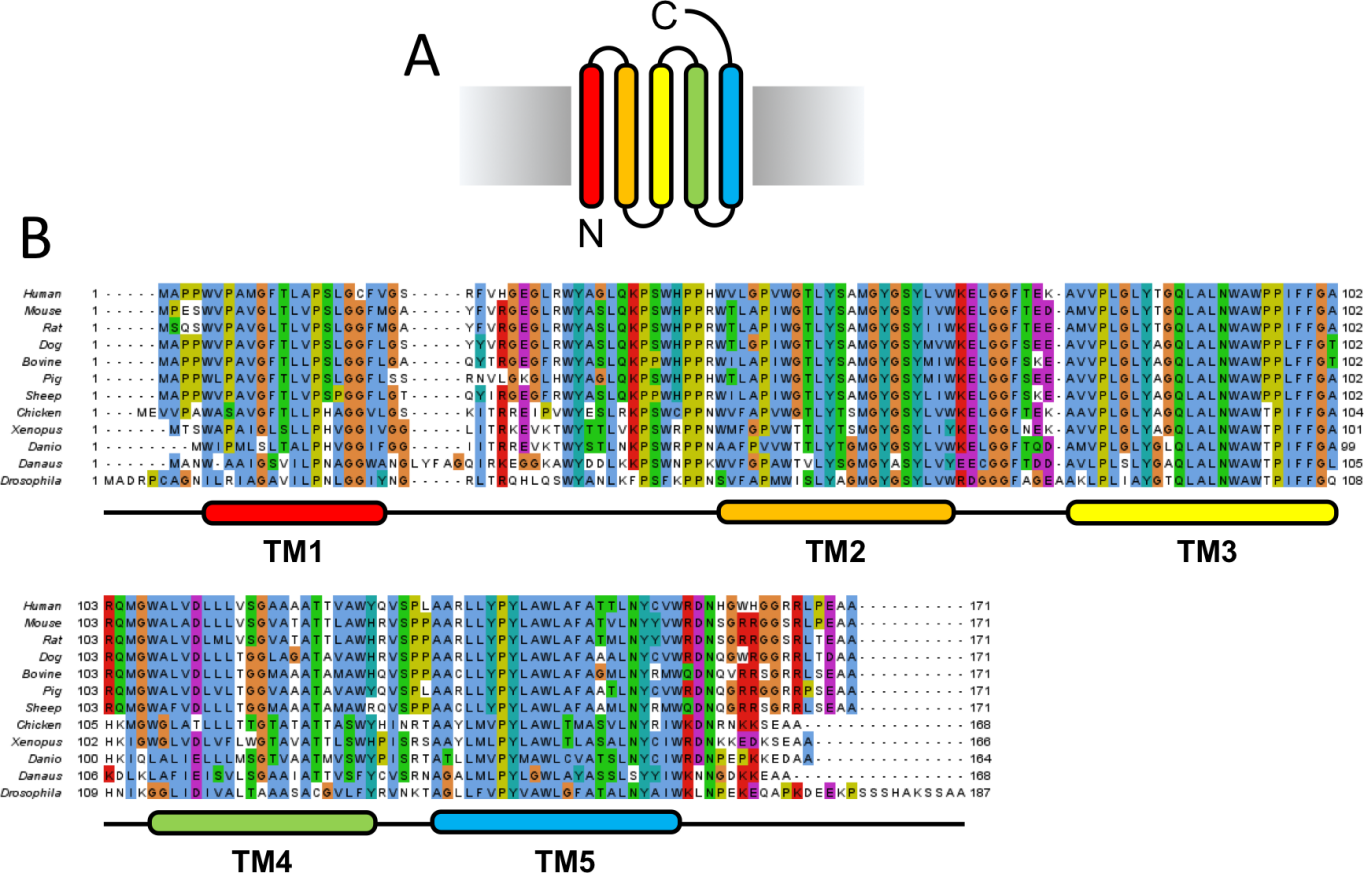
Sequence alignment of eukaryotic TSPO homologues. (A) TSPO topology in the membrane. (B) Multiple sequence alignment of eukaryotic TSPO homologues chosen for the initial expression test was performed using Jalview version 2.8.1.

The choice of a protein homologue that is used in a structural study can define the success of the subsequent structure determination project. To identify the most promising candidate proteins, we chose 12 eukaryotic TSPO homologues for an initial expression screen (Fig 1). Each of the proteins was tagged at the N- and C-terminus with a fluorescent protein (GFP) tag and a 10xHis-tag for subsequent affinity purification. Each construct was cloned into a mammalian expression vector and subjected to a small-scale expression test in HEK293T cells.

All 24 constructs could be expressed and detected by FSEC. The TSPO constructs tagged at the C-terminus expressed at 3- to 10-fold higher levels and were overall more monodisperse than the N-terminally tagged proteins. Like most mitochondrial proteins, TSPO is encoded in the nucleus and subsequently chaperoned and incorporated into the mitochondrial membrane by proteins of the mitochondrial protein import machinery [44]. Various regions of TSPO have been suggested to be involved in mitochondrial targeting [45]. It is possible that a bulky protein tag, such as GFP, introduced at the N-terminus may interfere with mitochondrial targeting and membrane insertion of TSPO, resulting in lower expression levels.

**Fig 2:**
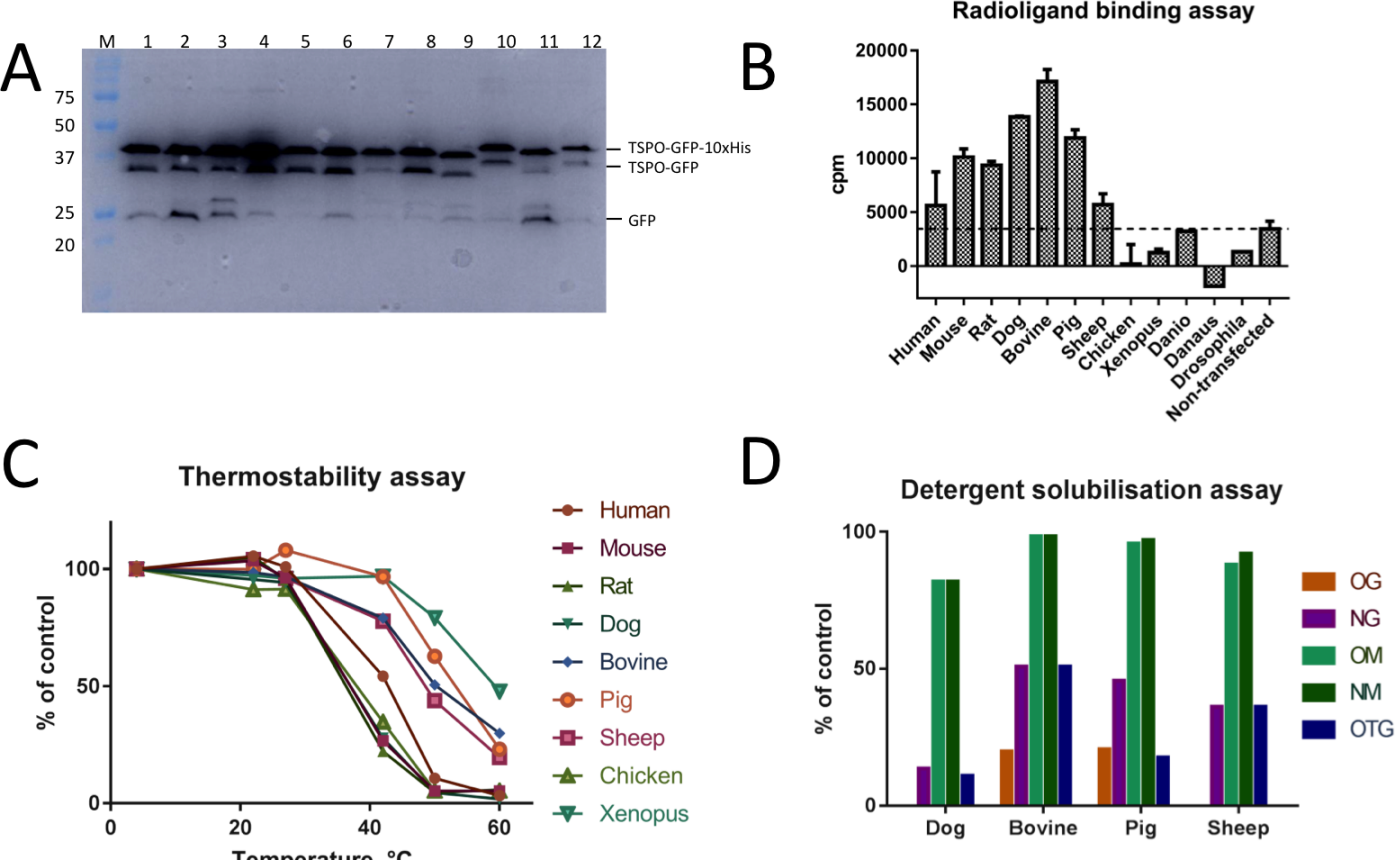
Overview of the screening procedures performed for homologue selection. (A) Small-scale expression test: in-gel fluorescence showing the expression levels of C-terminal GFP-10xHis constructs expressed in HEK293T cells and solubilised in 1% DM (1 human, 2 mouse, 3 rat, 4 dog, 5 bovine, 6 pig, 7 sheep, 8 chicken, 9 *Xenopus laevis*, 10 *danio rerio*, 11 *danaus plexippus*, 12 *drosophila melanogaster*). (B) Radioligand binding assay with [^3^H]PK11195 performed with all 12 C-terminally GFP-tagged TSPO homologues. (C) Thermostability of nine eukaryotic TSPO homologues assessed by incubating the in DDM solubilised proteins for one hour at a range of temperatures and subsequent analysis by FSEC. Control sample was incubated at 4°C (D) Small-micelle detergent screen of dog, bovine, pig and sheep TSPO. DDM-solubilised TSPO was diluted 10-fold into a buffer containing a secondary detergent, analysed by FSEC and the peak height was compared to the DDM-solubilised control sample.

The 12 C-terminally tagged constructs appeared as double bands on the fluorescence gel and most of them showed free GFP bands (Fig 2). We avoided heating the samples that were subjected to SDS-PAGE analysis to (a) prevent potential aggregation of the highly hydrophobic membrane protein, and (b) preserve GFP fluorescence. Therefore, it is likely that the double bands of TSPO fusions appeared due to partially retained secondary structure of the membrane protein that is incompletely denatured by SDS-containing loading buffer.

**Fig 3:**
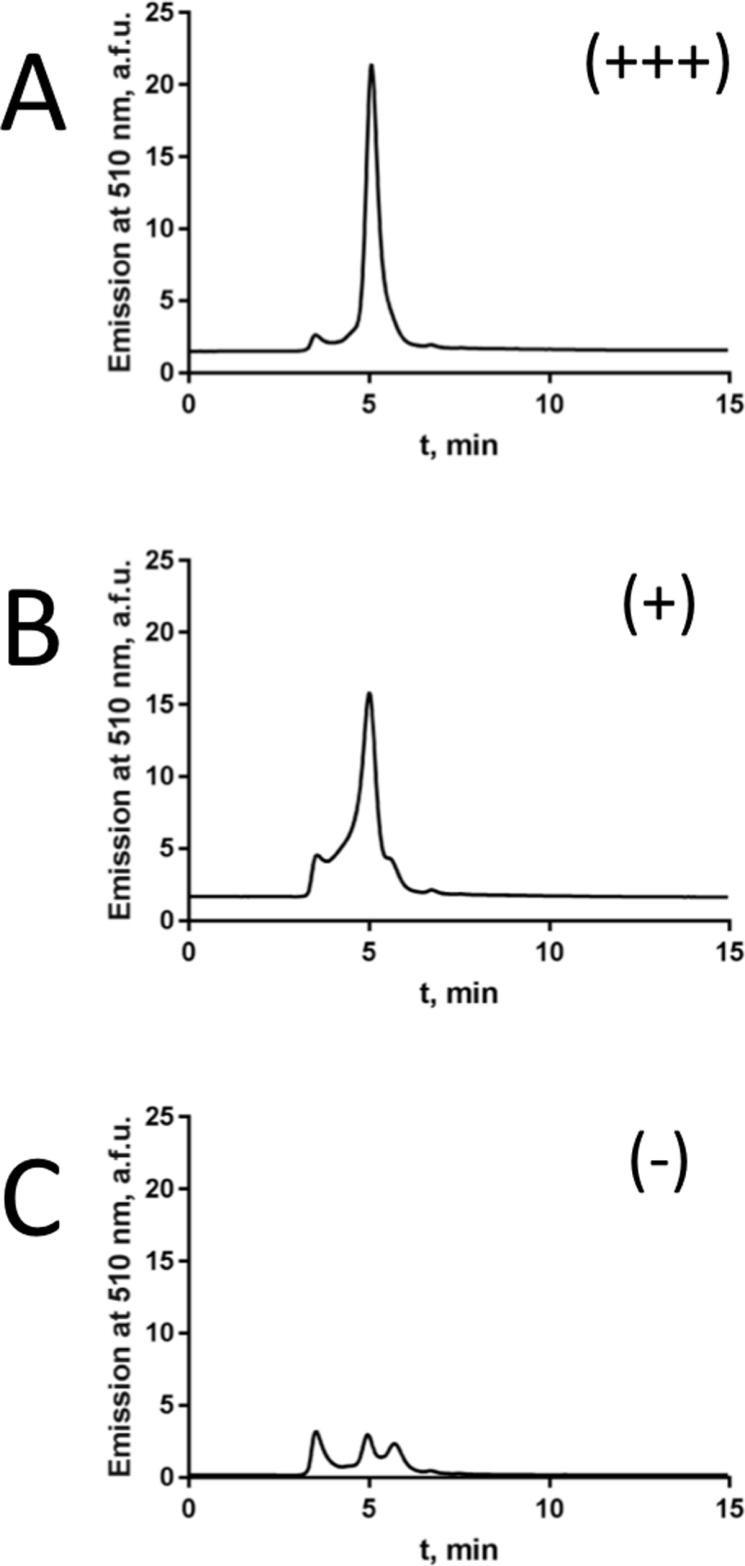
FSEC profiles of a representative TSPO homologue under stabilising and non-stabilising conditions. Sheep TSPO with C-terminal GFP-10xHis in DDM (A), DM (B), and with N-terminal GFP-10x-His in DDM (C) as an example for a monodisperse single peak (+++), a peak with a “shoulder” (+) and a low quality and/or polydisperse peak (-).

The detergent stability screen (Table 1 and Fig 3) showed that the homologues that were monodisperse in at least one detergent were those from human, dog, bovine, pig, sheep and chicken. The homologues from *danio rerio*, *danaus plexipus* and *drosophila melanogaster* were suboptimal in the initial screen, possibly due to differences in the membrane composition of the original host organisms corresponding to these proteins.

**Table 1:** Stability of TSPO in detergents.

	C-terminal GFP-10xHis				N-terminal GFP-10xHis	
Organism	DDM	LDAO	DM	C12E8	DDM	LDAO
Human	+	+++	-	+	-	+
Mouse	-	+	-	-	-	+
Rat	+	+	-	+	-	+
Dog	+++	+++	-	+++	-	+
Bovine	+++	+++	+	+	-	+
Pig	+++	+	+	+	+	+
Sheep	+++	+++	+	+	-	+
Chicken	+++	+++	-	+	+	-
Xenopus laevis	+	+	+	+	+	+
Danio rerio	-	-	-	-	-	-
Danaus plexippus	-	-	-	-	-	-
Drosophila melanogaster	-	-	-	-	-	-

Overview of the homogeneity of 12 TSPO homologues solubilised in common detergents and analysed by FSEC. The ranking was performed by assessment of the quality of the TSPO FSEC profile, focused on TSPO peak appearing in the elution time of 3.5–5 min on Agilent SEC5-300 HPLC column (as shown in Fig 3). Elution profiles were classified as those containing a monodisperse TSPO peak (+++), a peak with a “shoulder” (+) and a low quality and/or polydisperse peak (-).

All of the detergent-stable homologues, except chicken TSPO, showed binding to the radioactive analogue of PK11195 (Fig 2B). This suggested that the expressed proteins are well folded; we did not pursue a detailed characterisation of the radioligand binding profiles of the proteins, but instead used the binding assays as an effective screening tool for functional TSPO in the cellular membranes.

**Fig 4:**
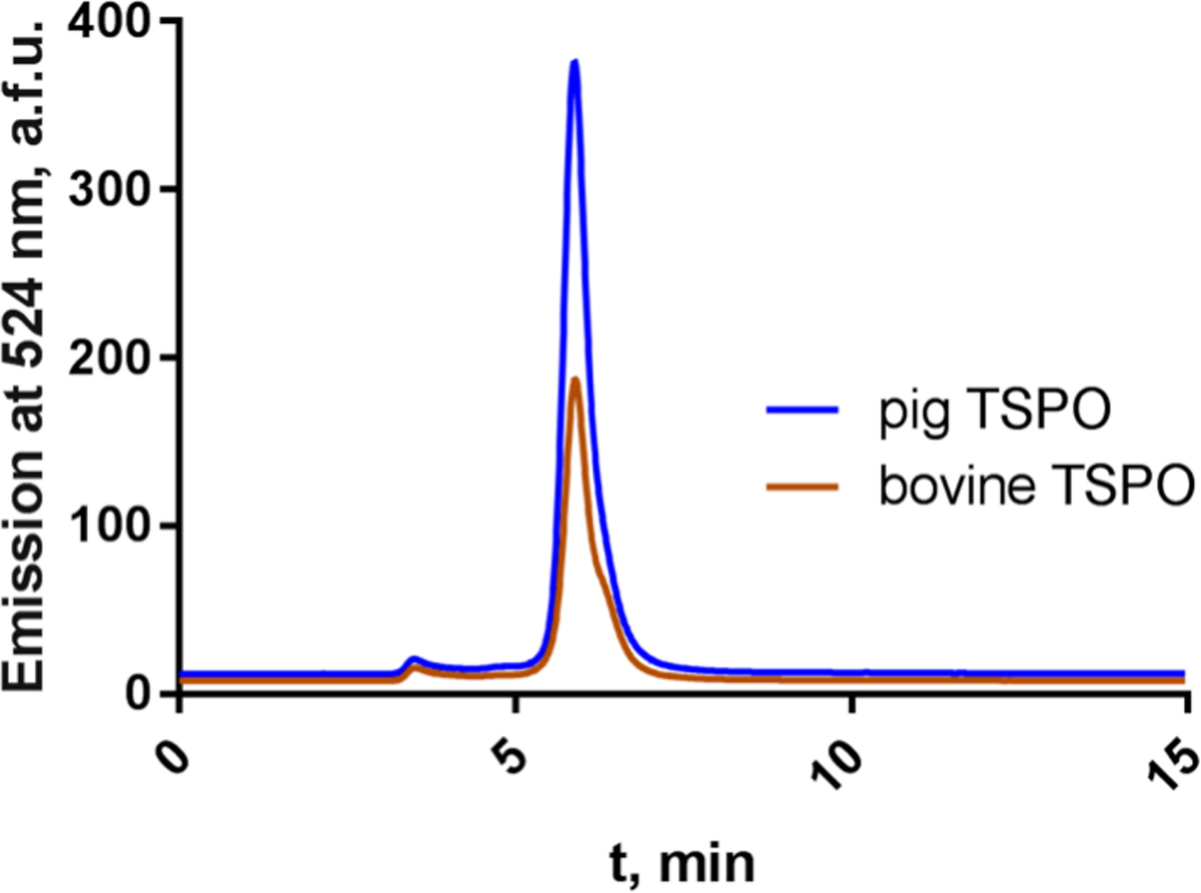
Stable HEK293 cell line expressing a cleavable TSPO. Comparison of the best clones of bovine and pig TSPO using FSEC. Normalised to total protein concentration with Bradford assay, determined using the cell lysates. Pig TSPO shows a 2-fold higher expression compared to bovine TSPO and was therefore chosen for subsequent large-scale expression.

The requirement to isolate the membrane proteins from the membrane environment for structural studies necessitates identifying the protein samples that retain their native-like state upon detergent extraction. We used an established FSEC-based technique [42, 46] to determine which of the TSPO homologues could proceed to the next stage of the protein production process. The thermostability assays using a commonly used mild detergent, DDM, highlighted dog, pig, bovine and sheep TSPO homologues as the most stable ones in detergent environment (Fig 2C). In many cases DDM is not the detergent of choice for crystallisation of membrane proteins, and it may be beneficial to exchange the detergent to another one with more beneficial properties, such as smaller micelle size [47, 48]. We therefore performed a secondary detergent screen with the four identified stable TSPO homologues, as detailed in”Materials and Methods” (Fig 2D). In this assay, the protein was tested for stability in small-micelle detergents, which can be more destabilising than DDM, but could be advantageous for structural characterisation of the protein later on. All four homologues were to some extent stable in NG, OM, NM and OTG, but only bovine and pig TSPO were stable in OG. Therefore, these two homologues were chosen for stable cell line generation. Our stable cell line generation efforts (Fig 4) revealed that the pig TSPO has substantially higher yields, compared to the bovine TSPO. Thus, we focus all subsequent experiments on the pig TSPO homologue.

### Linker engineering for fusion constructs

**Fig 5:**
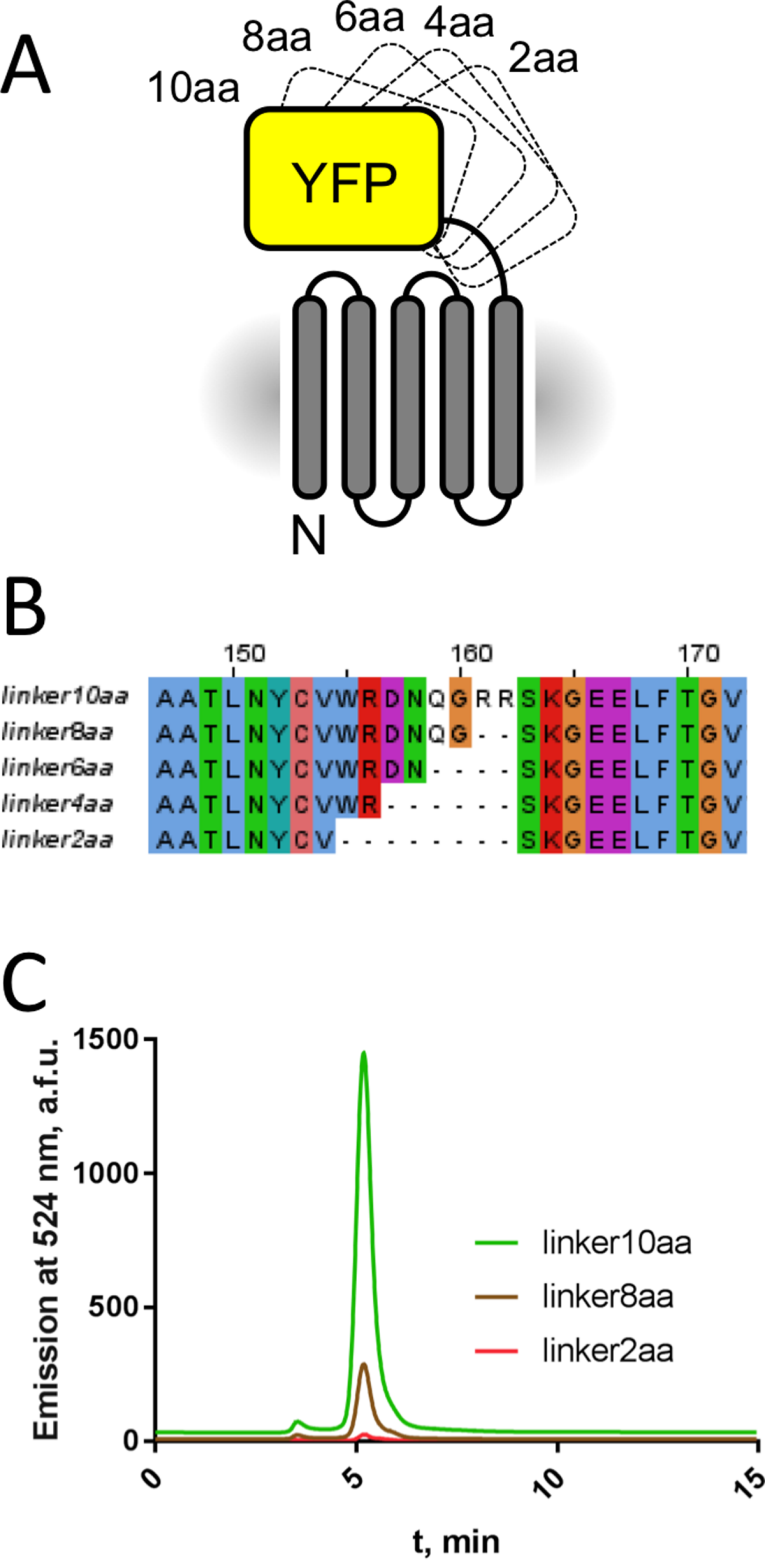
Optimisation of the TSPO-YFP fusion constructs. (A) Construct design for TSPO-YFP fusions. The linker between the proteins was shortened with two amino acid steps. (B): Sequence alignment of linker region for all five fusion constructs. (C) Expression level of the best clone of the TSPO-YFP fusion constructs after stable cell line generation analysed by FSEC.

The bulk of TSPO is embedded in the lipid bilayer. In some instances, the addition of solvent-exposed fusion proteins to the membrane protein of interest can facilitate its crystallisation by providing additional protein surfaces that can participate in crystallogenesis [34]. We engineered fusion constructs of TSPO with soluble proteins that are known to aid crystallisation of other membrane proteins: yellow fluorescent protein (YFP) and T4 lysozyme [49–51]. We engineered a small library of linkers (with a two-residue step) between the two fused parts to obtain stable constructs that can be used for downstream experiments (Fig 5A). Expression in HEK293S GnTi^-^ cells showed that the YFP fusions could be readily expressed (Fig 5B). In contrast, our attempts to express the lysozyme fusions resulted in cell death. Thus the linker engineering was performed only for the YFP fusions. All five fusion constructs could be expressed and for the three well-expressed protein fusions (linker10aa, linker8aa, linker2aa) stable cell lines were generated (Fig 5).

### Large-scale purification of pig TSPO and pig TSPO fusions

**Fig 6:**
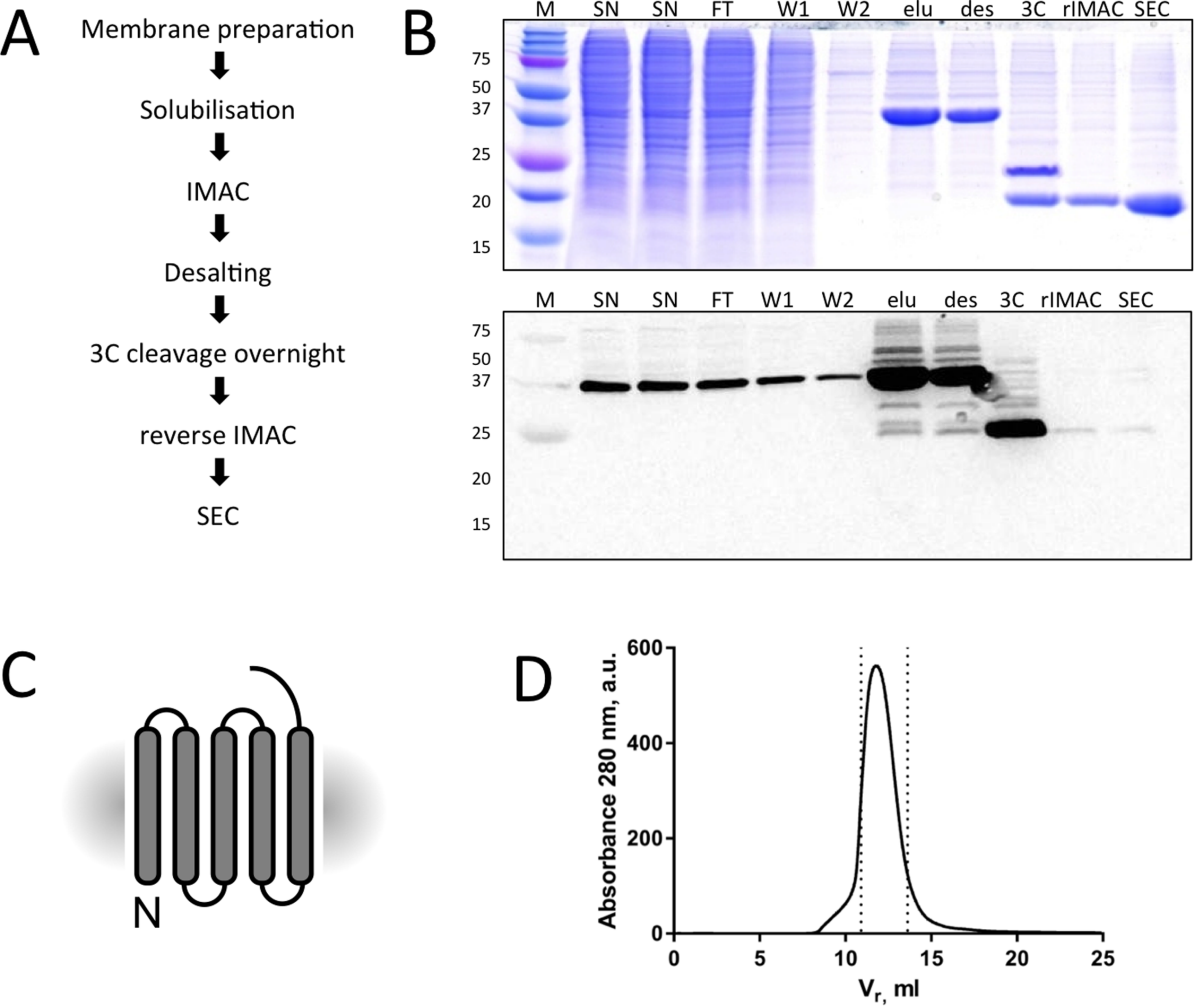
Large-scale purification of cleavable mammalian TSPO. (A) Overview of the purification steps. (B) Coomassie blue stained SDS-PAGE gel and in gel fluorescence of protein at various stages of purification. The lanes are labelled as follows: “M”—molecular weight marker, “SN”—supernatant, “FT” flow-through, “W1”—wash 1, “W2”—wash 2, “elu”–IMAC elution, “des”—desalting, “3C”–protein after 3C cleavage overnight, “rIMAC”—flow-through of the reverse IMAC procedure, “SEC”—pooled fractions after SEC. The final sample after SEC is monodisperse and does not contain any YFP. (C) Final construct after purification. (D) Size-exclusion profile of cleavable pig TSPO. The fractions of the peak taken for subsequent studies are indicated with the dashed lines.

**Fig 7:**
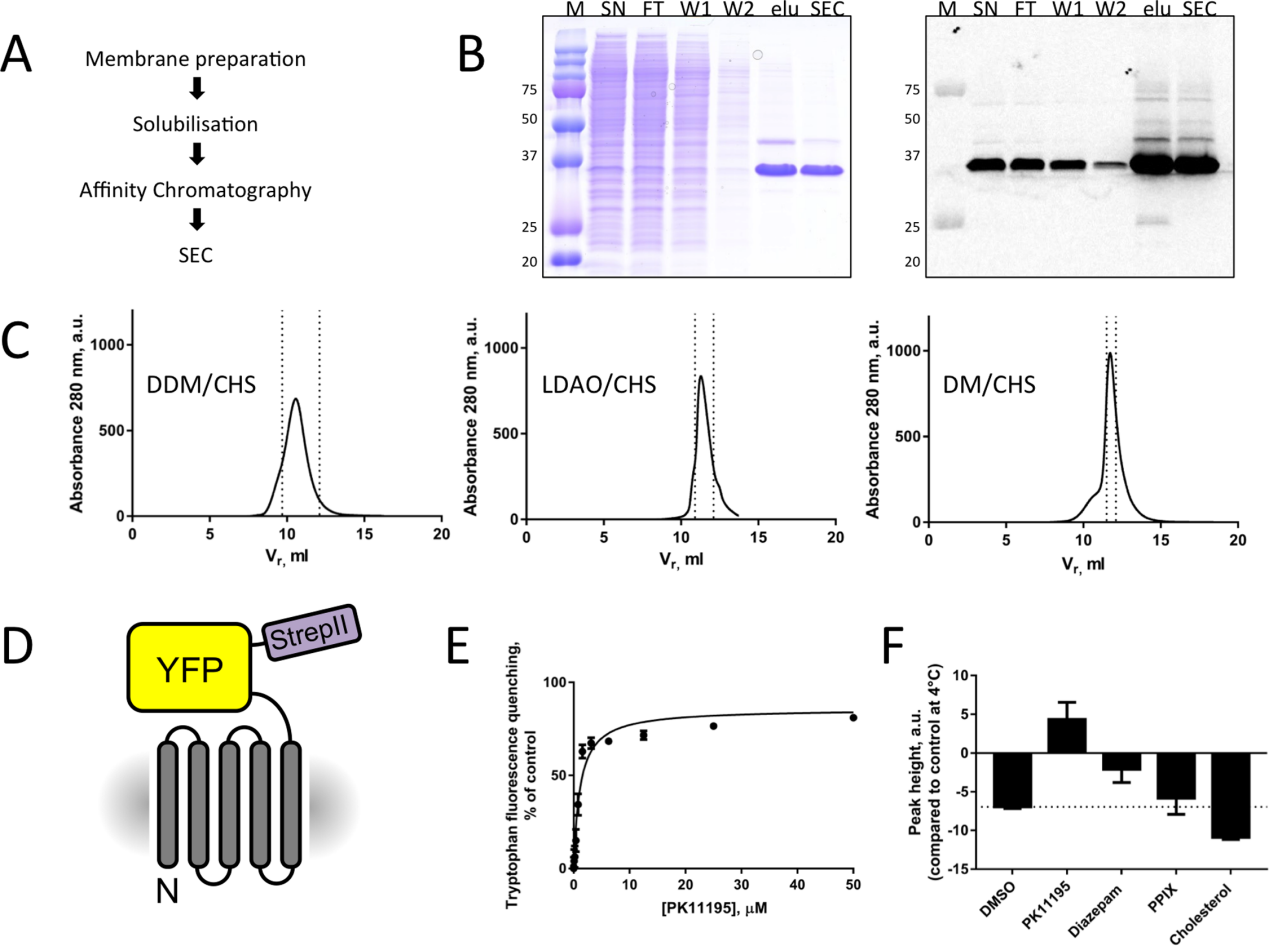
Large-scale purification of pig TSPO-YFP fusions. (A) Overview of the purification steps. (B) Coomassie blue stained SDS-PAGE gel and in gel fluorescence of protein at various stages of purification. The lanes are labelled as follows: “M”—molecular weight marker, “SN”—supernatant, “FT”—flow-through, “W1”—wash 1, “W2”—wash 2, “elu”–eluted fractions after affinity chromatography, “SEC”—pooled fractions after SEC. (C) Large-scale purification of TSPO-YFP fusion linker10aa in different detergents. Size-exclusion chromatography of pig TSPO fusion solubilised in DDM/CHS and subsequently purified in DDM/CHS (*left*), LDAO/CHS (*middle*) and DM/CHS (*right*). Peaks normalised to 25 g cells. (D) Final construct after purification. (E) Tryptophan quenching assay of TSPO-YFP with diazepam and PK11195 (n = 3). (F) Thermostability of the purified TSPO was assessed as described in “Materials and Methods”. Especially PK11195 has a stabilising effect on TSPO.

Using the optimised constructs identified in the small-scale experiments, we scaled up the protein expression to 4 L of mammalian culture, and performed large-scale purification of TSPO. Pure and homogenous protein could be obtained, following the optimised procedures detailed in “Materials and Methods”. The final yield of the pig TSPO was 0.15 mg per litre suspension culture and the protein could be concentrated to 10 mg/ml. The pig TSPO fusions had a 5-fold higher yield in comparison to the pig TSPO with removable YFP and could be concentrated to 20 mg/ml. An overview of the purification results is shown in Figs 6 and 7; normalized SEC data are provided in the Supporting Information.

Intrinsic tryptophan quenching assays with the known TSPO ligand PK11195 showed that the purified pig TSPO can bind PK11195 with an affinity in the low μM range (Fig 7E). The thermostability assay with the addition of drugs gives indications of which small molecules may be instrumental in stabilising the pig TSPO for future structural studies. The most prominent stabilising effect could be observed for PK11195 (Fig 7F).

The established procedure can routinely be used to generate stable and monodisperse YFP-tagged or untagged mammalian TSPO preparation of high purity and in quantities sufficient for structural studies. Furthermore, the obtained protein preparations are amenable to work under a variety of conditions, in the presence of detergents of varied chemical properties and micelle size, potentially facilitating future structural studies of TSPO.

## Discussion

We have established a protocol to produce a mammalian TSPO homologue in large scale in HEK293 cells. The stable homologue that we identified, pig TSPO, has a sequence identity of 84% to the human TSPO, and thus it can serve as an excellent model for the pharmacologically relevant human homologue. Future structural studies of pig TSPO, enabled by the procedures described here, may provide further insights in the function of this protein and thereby further explain its role in steroidogenesis and in TSPO-related diseases.

Previous biochemical and structural studies involved expression of a mammalian TSPO in *E*.*coli*, with subsequent protein extraction from the membranes using SDS [52], a harsh chaotropic detergent bound to interfere with the functionality of the protein. A similar approach, involving detergent substitution with dodecylphosphocholine, was more recently adopted for structural characterisation of a mammalian TSPO by NMR [32, 53]. In contrast to the methods used in these earlier studies, our protocol is advantageous as it allows for the use of a mild detergent (e.g. DDM) to extract the protein from the membrane, with a substantial yield of membrane protein purified under native conditions and avoiding a refolding step. The challenge in using the prokaryotic cells for mammalian membrane protein production may stem from several factors, including lipid bilayer composition, protein production and folding machinery and posttranslational modifications. In the case of mammalian TSPO homologues, expression in mammalian cells should ensure that it is produced in as close to a native environment as possible, maximising the chances of purifying the protein in a native conformation.

In conclusion, generation of pure recombinant membrane proteins of high quality in large scale is still a bottleneck for structural studies of these proteins. Our procedures for expression and purification of the pig TSPO can be readily extended to other TSPO homologues and to unrelated membrane proteins, provided the conditions for their stabilisation can be found. The methods described here will facilitate the detailed structural and biochemical characterisation of TSPO and other mammalian membrane proteins.

## Supporting information

S1 File Sequences of the TSPO constructs. Nucleotide and protein sequences of the key constructs used in the study: pigTSPO-3C-GFP-10xHis and pigTSPO-YFP-strepII.
(PDF)

S2 File Size exclusion chromatography data for the pig TSPO constructs. SEC data for proteins in DDM (for pigTSPO-3C-GFP-10xHis) or in DDM/CHS, LDAO/CHS and DM/CHS (for pigTSPO-YFP-strepII).
(XLSX)
