# Supplementary material for: Expression and purification of the mammalian translocator protein for structural studies
Source: PLoS One. 2018 Jun 13;13(6):e0198832. doi: 10.1371/journal.pone.0198832 (PMC5999236; doi:10.1371/journal.pone.0198832)
Supplement: S1 File — Nucleotide and protein sequences of the key constructs used in the study: pigTSPO-3C-GFP-10xHis and pigTSPO-YFP-strepII. (PDF) [file pone.0198832.s001.pdf]

Sequences of the TSPO constructs

pigTSPO-3C-GFP-10xHis

ATGGCCCCCCCTTGGCTGCCTGCCGTGGGCTTTACCCTGGTGCCCAGCCTGGGCGGCTTTCTGAGCAGCAGGA
ACGTGCTGGGCAAGGGCCTGCACTGGTATGCCGGCCTGCAGAAGCCTAGCTGGCACCCTCCCCACTGGACCCT
GGCTCCTATCTGGGGCACCCTGTACAGCGCCATGGGCTACGGCAGCTACATGATCTGGAAGGAGCTGGGCGG
CTTCAGCGAGGAGGCTGTGGTGCCTCTGGGCCTGTATGCCGGACAGCTGGCCCTGAATTGGGCCTGGCCCCCT
CTGTTCTTTGGCGCCAGACAGATGGGCTGGGCCCTGGTGGATCTGGTGCTGACCGGAGGAGTGGCTGCCGCT
ACAGCCGTGGCCTGGTACCAGGTCAGCCCTCTGGCTGCCAGGCTGCTGTACCCCTATCTGGCCTGGCTGGCCTT
TGCCGCCACCCTGAACTACTGCGTGTGGAGGGACAACCAGGGCAGGAGAGGCGGCAGAAGACCCAGCGAGG
CGGCCGCGGCCGCACTGGAGGTGCTGTTCCAGGGACCTGGCGGAGTGAGCAAGGGCGAGGAGCTGTTCACC
GGGGTGGTGCCCATCCTGGTCGAGCTGGACGGCGACGTAAACGGCCACAAGTTCAGCGTGTCCGGCGAGGG
CGAGGGCGATGCCACCTACGGCAAGCTGACCCTGAAGTTCATCTGCACCACCGGCAAGCTGCCCGTGCCCTGG
CCCACCCTCGTGACCACCTTCGGCTACGGCCTGCAGTGCTTCGCCCGCTACCCCGACCACATGAAGCAGCACGA
CTTCTTCAAGTCCGCCATGCCCGAAGGCTACGTCCAGGAGCGCACCATCTTCTTCAAGGACGACGGCAACTACA
AGACCCGCGCCGAGGTGAAGTTCGAGGGCGACACCCTGGTGAACCGCATCGAGCTGAAGGGCATCGACTTCA
AGGAGGACGGCAACATCCTGGGGCACAAGCTGGAGTACAACTACAACAGCCACAACGTCTATATCATGGCCG
ACAAGCAGAAGAACGGCATCAAGGTGAACTTCAAGATCCGCCACAACATCGAGGACGGCAGCGTGCAGCTCG
CCGACCACTACCAGCAGAACACCCCCATCGGCGACGGCCCCGTGCTGCTGCCCGACAACCACTACCTGAGCTA
CCAGTCCGCCCTGAGCAAAGACCCCAACGAGAAGCGCGATCACATGGTCCTGCTGGAGTTCGTGACCGCCGCC
GGGATCACTCTCGGCATGGACGAGCTGTACAAGGCTGCACACCATCACCATCACCATCACCATCACCATTAA

Protein sequence (TSPO indicated in red):

MAPPWLPAVGFTLVPSLGGFLSSRNVLGKGLHWYAGLQKPSWHPPHWTLAPIWGTLYSAMGYGSYMIWKELGG
FSEEAVVPLGLYAGQLALNWAWPPLFFGARQMGWALVDLVLTGGVAAATAVAWYQVSPLAARLLYPYLAWLAF
AATLNYCVWRDNQGRRGGRRPSEAAAAALEVLFQGPGGVSKGEELFTGVVPILVELDGDVNGHKFSVSGEGEGD
ATYGKLTLKFICTTGKLPVPWPTLVTTFGYGLQCFARYPDHMKQHDFFKSAMPEGYVQERTIFFKDDGNYKTRAEV
KFEGDTLVNRIELKGIDFKEDGNILGHKLEYNYNSHNVYIMADKQKNGIKVNFKIRHNIEDGSVQLADHYQQNTPIG
DGPVLLPDNHYLSYQSALSKDPNEKRDHMVLLEFVTAAGITLGMDELYKAAHHHHHHHHHH*

pigTSPO-YFP-strepII

ATGGCTCCTCCATGGCTGCCTGCCGTGGGCTTTACACTGGTGCCTAGCCTGGGCGGCTTCCTGAGCAGCAGAA
ACGTGCTGGGCAAGGGCCTGCATTGGTACGCCGGACTGCAGAAGCCTAGCTGGCACCCTCCTCACTGGACCCT
GGCCCCTATCTGGGGCACACTGTACAGCGCCATGGGCTACGGCAGCTACATGATCTGGAAAGAGCTGGGCGG
ATTCAGCGAAGAGGCCGTGGTGCCTCTGGGCCTGTATGCTGGACAGCTGGCCCTGAATTGGGCCTGGCCCCCT
CTGTTCTTTGGCGCCAGACAGATGGGATGGGCCCTGGTGGATCTGGTGCTGACAGGCGGAGTGGCCGCTGCT
ACAGCCGTGGCTTGGTATCAGGTGTCCCCTCTGGCCGCCAGACTGCTGTACCCTTATCTGGCCTGGCTGGCCTT
TGCCGCCACCCTGAATTACTGCGTGTGGCGGGACAACCAGGGCAGGCGGTCTAAGGGCGAGGAACTGTTTAC
CGGCGTGGTGCCCATCCTGGTGGAACTGGATGGCGACGTGAACGGCCACAAGTTCAGCGTGTCCGGCGAGGG
CGAAGGCGACGCCACATACGGAAAGCTGACCCTGAAGTTCATCTGCACCACCGGCAAGCTGCCCGTGCCTTGG
CCAACCCTCGTGACCACATTTGGCTACGGCCTGCAGTGCTTCGCCAGATACCCCGACCACATGAAGCAGCACG
ATTTCTTCAAGAGCGCCATGCCCGAGGGCTACGTGCAGGAACGGACCATCTTCTTCAAGGACGACGGCAACTA
CAAGACCAGAGCCGAAGTGAAGTTCGAGGGCGACACCCTCGTGAACCGGATCGAGCTGAAGGGCATCGACTT

CAAAGAGGATGGCAACATCCTGGGCCACAAGCTGGAGTACAACTACAACAGCCACAACGTGTACATCATGGC
CGACAAGCAGAAAAACGGCATCAAAGTGAACTTCAAGATCCGGCACAACATCGAGGACGGCAGCGTGCAGCT
GGCCGACCACTACCAGCAGAACACCCCCATCGGAGATGGCCCCGTGCTGCTGCCCGACAACCACTACCTGAGC
TACCAGAGCGCCCTGAGCAAGGACCCCAACGAGAAGCGGGACCACATGGTGCTGCTGGAATTTGTGACCGCC
GCTGGCATCACCAGCGCCTGGTCCCACCCTCAGTTCGAGAAGTGA

Protein sequence (TSPO indicated in red):

MAPPWLPAVGFTLVPSLGGFLSSRNVLGKGLHWYAGLQKPSWHPPHWTLAPIWGTLYSAMGYGSYMIWKELGG
FSEEAVVPLGLYAGQLALNWAWPPLFFGARQMGWALVDLVLTGGVAAATAVAWYQVSPLAARLLYPYLAWLAF
AATLNYCVWRDNQGRRSKGEELFTGVVPILVELDGDVNGHKFSVSGEGEGDATYGKLTLKFICTTGKLPVPWPTLV
TTFGYGLQCFARYPDHMKQHDFFKSAMPEGYVQERTIFFKDDGNYKTRAEVKFEGDTLVNRIELKGIDFKEDGNILG
HKLEYNYNSHNVYIMADKQKNGIKVNFKIRHNIEDGSVQLADHYQQNTPIGDGPVLLPDNHYLSYQSALSKDPNEK
RDHMVLLEFVTAAGITSAWSHPQFEK*
